# Gulf War Agent Exposure Causes Impairment of Long-Term Memory Formation and Neuropathological Changes in a Mouse Model of Gulf War Illness

**DOI:** 10.1371/journal.pone.0119579

**Published:** 2015-03-18

**Authors:** Zuchra Zakirova, Miles Tweed, Gogce Crynen, Jon Reed, Laila Abdullah, Nadee Nissanka, Myles Mullan, Michael J. Mullan, Venkatarajan Mathura, Fiona Crawford, Ghania Ait-Ghezala

**Affiliations:** 1 The Roskamp Institute, Sarasota, Florida, United States of America; 2 The Open University, Walton Hall, Milton Keynes, Buckinghamshire, United Kingdom; 3 James A. Haley Veteran’s Hospital, Tampa, Florida, United States of America; 4 University of Miami Miller School of Medicine, Miami, Florida, United States of America; University of Insubria, ITALY

## Abstract

Gulf War Illness (GWI) is a chronic multisymptom illness with a central nervous system component such as memory deficits, neurological, and musculoskeletal problems. There are ample data that demonstrate that exposure to Gulf War (GW) agents, such as pyridostigmine bromide (PB) and pesticides such as permethrin (PER), were key contributors to the etiology of GWI post deployment to the Persian GW. In the current study, we examined the consequences of acute (10 days) exposure to PB and PER in C57BL6 mice. Learning and memory tests were performed at 18 days and at 5 months post-exposure. We investigated the relationship between the cognitive phenotype and neuropathological changes at short and long-term time points post-exposure. No cognitive deficits were observed at the short-term time point, and only minor neuropathological changes were detected. However, cognitive deficits emerged at the later time point and were associated with increased astrogliosis and reduction of synaptophysin staining in the hippocampi and cerebral cortices of exposed mice, 5 months post exposure. In summary, our findings in this mouse model of GW agent exposure are consistent with some GWI symptom manifestations, including delayed onset of symptoms and CNS disturbances observed in GWI veterans.

## Introduction

At least 26–32% of US and UK military personnel who were deployed to the Persian Gulf in the 1990–91 conflict are currently afflicted with the chronic multi-symptom illness known as Gulf War Illness (GWI) [1–6]. Veterans with GWI exhibit persistent health issues such as fatigue, gastrointestinal problems, idiopathic pain, musculoskeletal problems, and neurological symptoms, with memory problems being one of the most commonly reported symptoms [2,5,7,8]. To date, there are no effective treatments for GWI, and thus identification of biological pathways associated with long-term GWI sequelae is vital to determining the pathogenic mechanisms for the development of novel therapies for the treatment of this devastating illness, which still afflicts our military population from that time period.

The multi-symptom clinical presentation associated with GWI is unique to the 1990–91 deployment, with no such illness being reported in any other military campaign, indicating that GWI etiology cannot solely be attributed to combat-related stress [2,9–11]. There are ample data to suggest that combined exposure to pyridostigmine bromide (PB) and permethrin (PER), referred to herein as “GW agents,” were key contributors to the etiology of GWI [1,12]. Additionally, other chemicals that may induce GWI symptoms have also been proposed, such as depleted uranium, multiple vaccinations against anthrax and botulinum, as well as exposure to low levels of nerve gas agents, including soman, sarin, and mustard gas [1,13–18]. This is supported by animal studies of GW agent exposure detailing the consequences of combined exposure to various agents that demonstrated sensorimotor deficits, altered brain acetylcholine (ACh) receptor binding, and increased activation of astrocytes in the brains of exposed animals [19–21]. However, it remains unclear which neuropathological manifestations may be responsible for the neurological symptoms observed in GWI patients.

We hypothesized that co-administration of PB and PER in our mouse model of GW agent exposure would recapitulate the late-onset symptom multiplicity and heterogeneity of symptoms observed in GW veterans, such as memory deficits and neurological deficits. To that end, we previously established a model of GWI in a CD1 mouse strain, which demonstrated impairment of long-term memory formation following acute (10 days) exposure to PB and PER [21].

In our current study, we expanded upon our previous investigation in order to explore the timing of symptoms and the relationship between memory deficits and neuropathological changes associated with GW agent exposure. Furthermore, we translated our previous exposure paradigm to the more common C57BL6/J strain, which is one of the most widely used and well- characterized inbred strains, in order to facilitate replication and further development of this model by the GWI research community. We postulate that such functional characterization of the pathobiology associated with GW agent exposure will contribute to our understanding of the molecular mechanisms that lead to GWI.

## Experimental Procedures

### Gulf War Chemical Agents

Pyridostigmine bromide (PB) (99.4%) was purchased from Fisher Scientific (Hanover Park, IL), and permethrin (PER) (98.3% mixture of 27.2% cis and 71.1% trans isomers) was purchased from Sigma Aldrich (St. Louis, MO). As there is no information currently available on the exact cis/trans ratio of PER that was used in the 1990–1991 Gulf War, we used this commercially available ratio since it was similar to that recommended by the World Health Organization (25% cis and 75% trans) [22]. For the Short-term and the Long-term Cohorts, we used 200 mg/kg of PER and 0.7 mg/kg of PB, doses that have been used in previous mouse studies showing adverse behavioral or pathological outcomes [21,23,24]. These doses are less than one fifth and less than half of the reported mouse LD_50_ doses respectively [25,26].

### Animals

All animal experiments were approved by the Roskamp Institute’s Institutional Animal Care and Use Committee and conducted in accordance with the Office of Laboratory Animal Welfare and the Association for the Assessment and Accreditation of Laboratory Animal Care. Mice were purchased from Jackson Laboratories (Bar Harbor, Maine) and each mouse was individually housed in a controlled environment (regulated 14-h day/10-h night cycle) and maintained on a standard diet.

Forty-eight male C57BL6/J mice (12 weeks of age) were co-administered with either a 50 μl total volume of GW agents to a final dose of 0.7 mg/kg of PB and 200 mg/kg of PER in 100% dimethyl sulfoxide (DMSO) [exposed mice; n = 24], or a 50 μl volume of vehicle (100% DMSO) [control mice; n = 24] via intraperitoneal injection (IP) injection daily, for 10 days [21,24]. The mice were allowed to rest for an additional 10 days, and were then subjected to neurobehavioral testing. Mice (n = 48) were subjected to Barnes Maze testing 11–15 days post exposure, and the Short-term Cohort (n = 20) was euthanized shortly after the last probe trial day (18 days post exposure). The Long-term Cohort (n = 28) was assessed by probe trial every 30 days thereafter and euthanized when the late-onset of impairment in long-term memory formation was detected, approximately 5 months post exposure to GW agents.

### Neurobehavioral testing

All neurobehavioral testing was performed during the light phase of the circadian cycle with operators blinded to the exposure assignment. All trials were recorded and analyzed with the Ethovision tracking system (Noldus, Wageningen, Netherlands). The nice were randomized to be in either the Short-term or Long-term Cohorts prior to initiation of any studies using the Excel number generator. Thus, there was no chance of bias in the selection process between cohorts.

The Barnes Maze (BM) protocol was used to assess the spatial memory and learning for the Short and Long-term Cohorts [27,28]. The BM acquisition trials were conducted 11–14 days post-exposure, during which four trials were conducted per mouse daily for four days. In order to assess short-term spatial working memory, a single probe trial was carried out 15 days post-exposure. A subset of pre-randomized mice (exposed mice; n = 10, and control mice; n = 10) were euthanized at the end of the behavioral study in order to assess the short-term consequences of 10 days post-exposure; this cohort shall be referred to as the “Short-term Cohort” from now on. In order to assess the long-term consequences of 10 days exposure, the second subset of mice (exposed mice; n = 14, and control mice; n = 14) were assessed by probe trial every 30 days thereafter on post-exposure days 56, 77, and 106. Henceforth, this cohort shall be referred to as the “Long-term Cohort.”During the four days of acquisition trials (4 trials / mouse / day), each mouse was placed in the middle of the maze and allowed to explore the maze for a fixed interval of 180 seconds. The escape box was placed underneath the Target Hole (TH), and the mouse was allowed to escape and rest in the chamber for the duration of the acquisition trial. On the probe trial day (5^th^ day), the escape box was removed, and each mouse was then placed in the middle of the maze and allowed to explore the maze for a fixed interval of 90 seconds. The number of nose pokes in the target hole (frequency), the number of nose pokes into the holes other than the target hole (primaryerror rate) and the TH duration (the time(s) the mouse spent at the target hole), as well as the distance to TH (cm) (distance travelled to reach the virtual target hole), were among the dependent variables that were measured during the experiment as outcome factors.

#### Statistical analyses for neurobehavioral testing

The Barnes Maze data were analyzed using SPSS 21.0 (IBM corp., Armonk, NY). A mixed linear model (MLM) regression was employed to examine the independent effects of exposure and time and any potential interactions between them on the neurobehavioral outcomes [cumulative distance to TH (cm), latency (s), primary error rate (#), frequency (#) and velocity (cm/s)] for the Short-term and the Long-term Cohort. The MLM-based regression analysis approach is generally considered advantageous over other ANOVA due to its flexibility to accommodate fixed and random effects of the independent variables as well as incorporate dichotomous, continuous and categorical variables [29]. However, if the data were not normally distributed, we used a generalized linear model (GLM) to perform the analyses and evaluated non-parametric dependent variables using the Wald test. The Wald test can be used to test the true value of the parameter based on the sample estimate [30]. When examining the probe trial data from the Long-term Cohort, the dependent variables, frequency (#) and duration at TH (s), were not normally distributed thus we used a generalized linear model (GLM) to perform the analyses. Statistical significance was set at the alpha 0.05 level for all statistical analysis. All graphs are depicted as means, and error bars show standard error of the mean (SEM).

### Immunohistochemical procedures

The left hemisphere from all animals was immersed in 4% Paraformaldehyde (PFA) for 24 hours and paraffin embedded. Six μm thick sagittal sections were deparaffinized and rehydrated in an ethanol gradient before starting each procedure. Four sets of sagittal sections per animal and a minimum of 8 animals per group from the Short-term and the Long-term Cohorts were used for each study.

#### Histological staining

Nissl staining was performed in order to examine the morphology and pathology of neuronal tissue. Briefly, sections were stained with 0.25% Cresyl Violet for 20 minutes, then quickly rinsed with water. The *in situ* cell death detection kit (Roche Diagnostics, Indianapolis, IN) was used for terminal deoxynucleotidyl transferase dUTP nick end labeling (TUNEL) staining, following manufacturer’s instructions. Labeling was performed with 3,3'Diaminobenzidine (DAB) as the chromogen. For the negative control, the enzyme solution was omitted from the TUNEL reaction mixture, while for the positive control, the slide was subjected to DNAse I (1000 U/ml) for 10 minutes at room-temperature to induce DNA strand breaks (as per manufacturer’s recommendations), prior to labeling procedures. Bielschowsky's silver staining was also used to check for the presence of degenerating neurons and damaged axons, as per manufacturer’s instructions (HitoBiotec Inc., Wilmington, DE).

#### Immunohistochemical staining

Glial fibrillary acidic protein antibody (GFAP, 1:10000, Dako, Carpinteria, CA) and ionized calcium binding adaptor molecule 1 antibody (IBA-1, 1:1000, Abcam, Cambridge, MA) were used to stain for astrocytes and for both activated and resting microglia / macrophages, respectively. Rat anti-mouse CD45 antibody (1:100, Serotec, Raleigh, NC) was used to stain for activated microglial cells. Pre-synaptic vesicles were detected using anti-synaptophysin antibody (SYP, 1:400, Abcam, Cambridge, MA). Tissue sections were subjected to heat-induced antigen retrieval using target retrieval solution, citrate buffer pH 6 (Dako, Carpinteria, CA) for IBA-1 and SYP IHC procedures. Endogenous peroxidase activity was quenched with H_2_O_2_ treatment (0.3% in water). Each section was rinsed and incubated with the appropriate blocking buffer (DAKO Serum Free Protein Block), before applying the appropriate primary antibody overnight at 4°C. Then, the diluted biotinylated secondary antibody from the ABC Elite Kit (VECTASTAIN Elite ABC Kit, Vector Laboratories, Burlingame, CA) was applied. The stain was developed using DAB peroxidase substrate solution and counterstained with hematoxylin. All IHC slides were dehydrated through the ethanol gradient, treated with xylene, before using permanent mounting medium to coverslip. Both microscopy and quantification were performed with the operator blinded to the exposure assignment.

#### Immunohistochemical image analysis

Briefly, non-overlapping RGB (red, green, blue) images were digitally captured within the defined areas of interest (hippocampi, including the dentate gyri, and the CA3 regions, as well as the cerebral cortices, from exposed and control mice, respectively) and were then optically segmented and analyzed as previously described by Schindelin *et al*. [31] using the FIJI open-source platform for biological image analysis (http://fiji.sc/Fiji).

#### Statistical Analysis for immunoreactivity of stained tissues

The immunoreactivity (percent area) of tissue stained with DAB was calculated by dividing the obtained mean RGB value (of segmented profiles) by the total RGB value per defined field, multiplied by 100. Data were separately plotted as the mean percentage area of immunoreactivity per field (denoted “% Area”) ± SEM for each region and grouping. Since we have carried out a variety of different histological analyses in the hippocampus (one of our regions of interest), we were limited to two randomly separate pairs of sagittal brain sections through the dorsal hippocampus, and thus were unable to perform stereological analyses. Student’s t-test was used if the outcome variable was normally distributed; otherwise an adaptation of the Student’s t-test was used. Welch’s t-test was utilized if the samples had unequal variances. Statistical analysis was performed using JMP 10.0 (SAS, Cary, NC). All graphs are depicted as means, and error bars show standard error of the mean (SEM).

### Quantification of ACh levels from brain homogenates

Determination of acetylcholine (ACh) levels was performed as previously described by Ojo et al [23], with slight modifications. Briefly, 45 μl of PBS fraction of the brain homogenate from the right hemisphere was combined with 5 μl of 750 ng/mL ACh-D4 (CDN Isotopes, Quebec, Canada) internal standard in 100% Mass Spectrometry (MS) grade acetonitrile (ACN), followed by an addition of 200 μl of ice-cold MS grade ACN to each sample, which was then centrifuged for 10 min at 15,800 × g. The supernatant for each sample was subsequently transferred to individual glass vials (Restek, Pennsylvania, US) and used for MS analysis.

ACh levels were analyzed by direct infusion MS. Chip-based nanospray was used to introduce each sample into an LTQ-Orbitrap mass spectrometer (Thermo, Waltham, MA, USA) via a Nanomate Triversa (Ithaca, NY, Advion). Acetylcholine and ACh-D4 precursor molecular ions, 146.12 m/z and 150.14 m/z respectively, were isolated simultaneously (isolation width = 10) in the ion trap, and fragmented using higher energy collisional dissociation (HCD) in the C-trap (relative collision energy = 77). Fourier transform mode (FTMS) at 100,000 resolution (m/z = 400) was used to acquire MS/MS spectra. Injections lasted for 30 sec, and five technical replicates were performed for each sample. Acetylcholine levels were calculated from the peak height ratios of D_0_ and D_4_ fragment ions (87.04 m/z for ACh-D_0_ and 91.07 m/z for ACh-D_4_) for each of the five replicates. Concentrations of ACh levels in each sample were calculated in reference to the concentration of the ACh-D_4_ internal standard, which were then normalized to the total protein concentration of each respective sample. Statistical analyses of ACh data were performed with mixed linear model (MLM) regression analyses using SPSS 21.0 (IBM corp. Armonk, NY).

## Results

### Neurobehavioral examination

**Fig 1:**
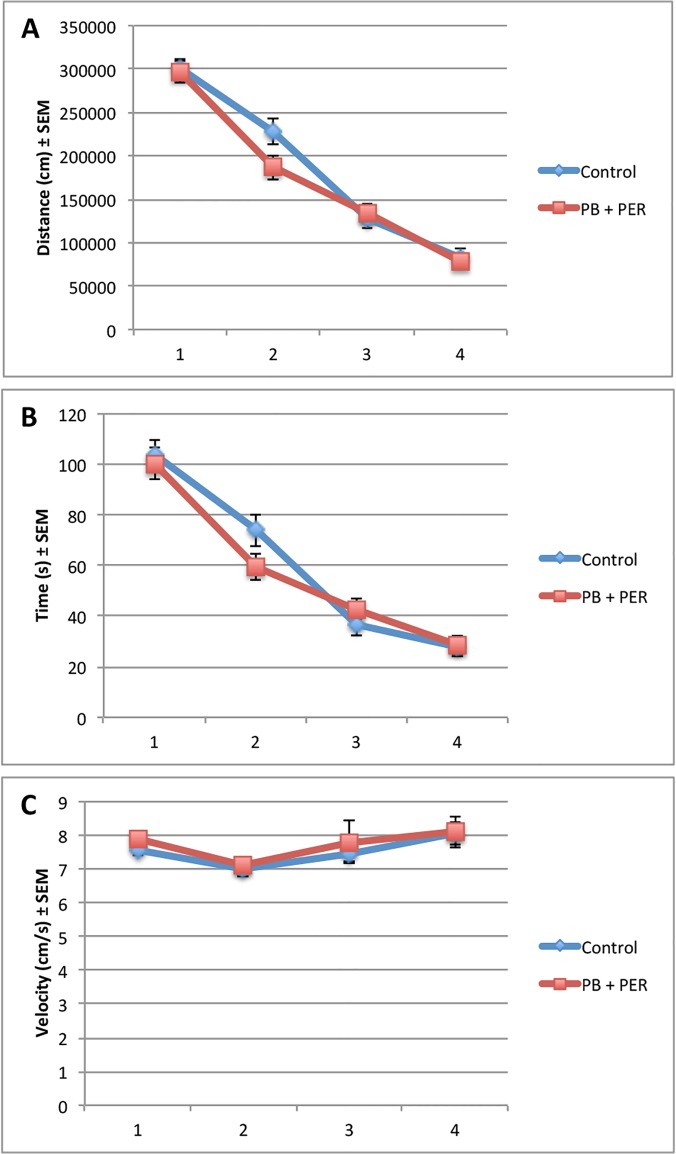
No overall behavioral differences were observed in exposed mice, 11–14 days post-exposure to PB + PER. Control and exposed mice from the Short-term and Long-term Cohorts (n = 48) behaved similarly when we examined (A) cumulative distance traveled to the target hole (F = 0.94, DF = 1, 167, p = 0.33), (B) escape latency (F = 0.32, DF = 1, 167, p = 0.57), and (C) velocity (F = 0.50, DF = 1, 148, p = 0.48), over a 4-day period. The data sets for Cumulative Distance to TH (cm), Escape latency (s), and Velocity (cm/s) were normally distributed. Therefore, a Mixed Linear Model (MLM) regression was run using SPSS 21.0 software. All graphs are depicted as means and error bars show standard error of the mean (SEM).

**Fig 2:**
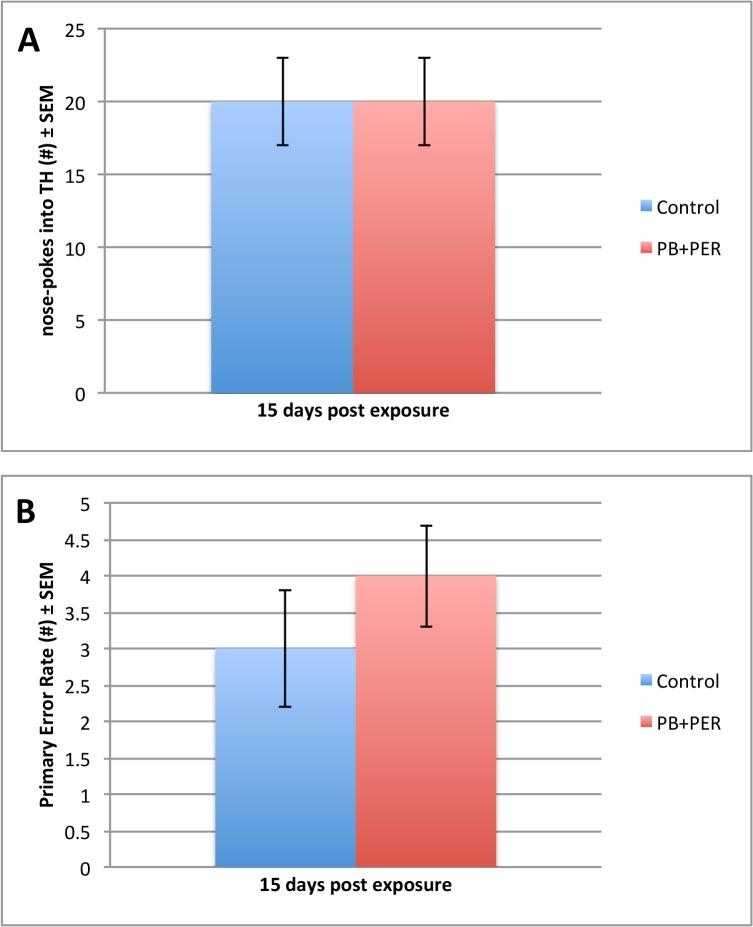
Short-term memory formation was unaffected 15 days after exposure to PB + PER. Frequency (#) and Primary Error Rate (#) were examined for exposed and control mice during Barnes Maze probe trials. For the frequency of nose-pokes into the target hole (A), no main effect of exposure was observed (F = 0.034, DF = 1,1, p = 0.86). For the primary error rate, there was no main effect of exposure (F = 0.46, DF = 1, 74.36, p = 0.50) that was observed during probe trials. SPSS 21.0 was used to test the true value of the parameter based on the sample estimate. All graphs are depicted as means and error bars show standard error of the mean (SEM).

**Fig 3:**
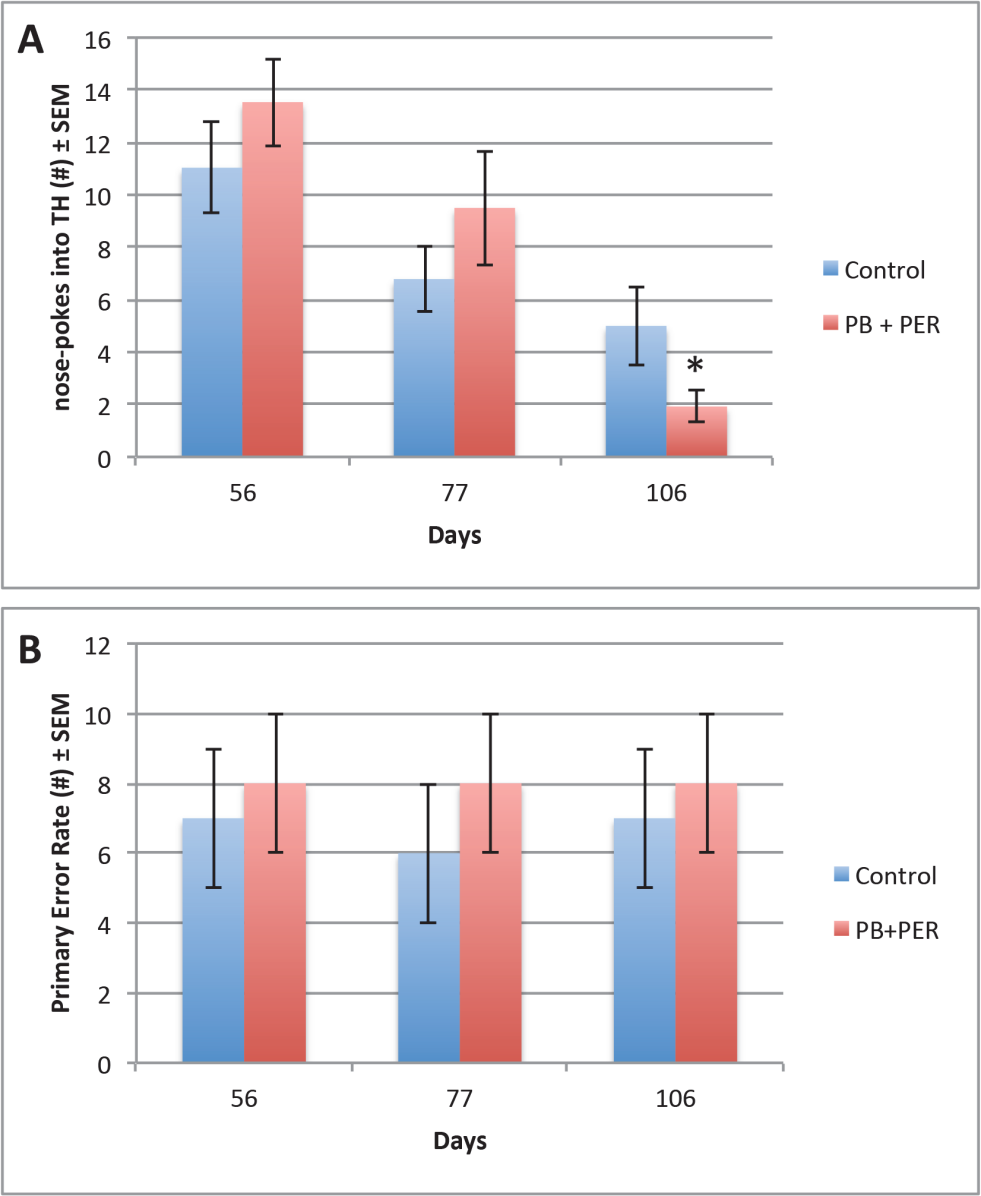
Impairment for long-term memory formation was observed at day 106 days-post acute exposure to PB + PER. Frequency (#) and Primary Error Rate (#) were examined for exposed and control mice during Barnes Maze probe trials. For the frequency of nose-pokes into the target hole (A), we observed a significant interaction between the exposure and days post-exposure (Wald = 6.15, df = 2, p = 0.05), a main effect of post-exposure days (Wald = 28.67, df = 2, p < 0.001) and no main effect of exposure (Wald = 0.94, df = 1, p = 0.33). For the primary error rate, there was no main effect of exposure (F = 0.85, DF = 1, 74.29, p = 0.36), and no interaction effect between the exposure and days post-exposure (F = 0.10, DF = 1, 52.27, p = 0.91) that was observed during probe trials. SPSS 21.0 was used to test the true value of the parameter based on the sample estimate. All graphs are depicted as means and error bars show standard error of the mean (SEM).

Data from the acquisition trials (days 11–14 post-exposure), showed that both the exposed and control mice (n = 48) performed similarly when cumulative distance traveled to the target hole (F = 0.94, DF = 1, 167, p = 0.33), escape latency (F = 0.32, DF = 1, 167, p = 0.57), and velocity (F = 0.50, DF = 1, 148, p = 0.48), over a 4-day period were assessed (Fig. 1). On day 5 of behavioral testing, the probe trial was conducted (15 days post-exposure). No differences were detected between the exposed and control mice (n = 24 per group) during probe trial (Fig. 2). The “Short-term Cohort,” a subset of the mice from the entire study (n = 10/group) were euthanized shortly after the probe trial day (18 days post exposure to GW agents). When examining the probe trial data from the Long-term Cohort, the dependent variables, frequency (#), primary error rate (#) and duration at TH (s), were not normally distributed, thus we used a generalized linear model (GLM) to perform the analyses. These non-parametric dependent variables were evaluated using the Wald test, thus allowing to test for the effect of post-exposure day as well as interaction of treatment with post-exposure days on the outcomes [30]. Additional probe trials were conducted at approximately 30-day intervals (day 56, 77 and 106 post-exposure) using the remaining animals (n = 28, the “Long-term Cohort”). When examining the frequency of nose-pokes into the target hole for the Long-term Cohort, we observed a significant interaction between the exposure and days post-exposure (Wald = 6.15, DF = 2, p = 0.05, see Fig. 3), a main effect of post-exposure days (Wald = 28.67, DF = 2, p < 0.001) and no main effect of exposure (Wald = 0.94, DF = 1, p = 0.33), indicating impairment of long-term memory formation, in which the exposed mice visited the target hole less frequently as compared to their controls over time.

### Neuropathological findings in the Short-term Cohort

**Fig 4:**
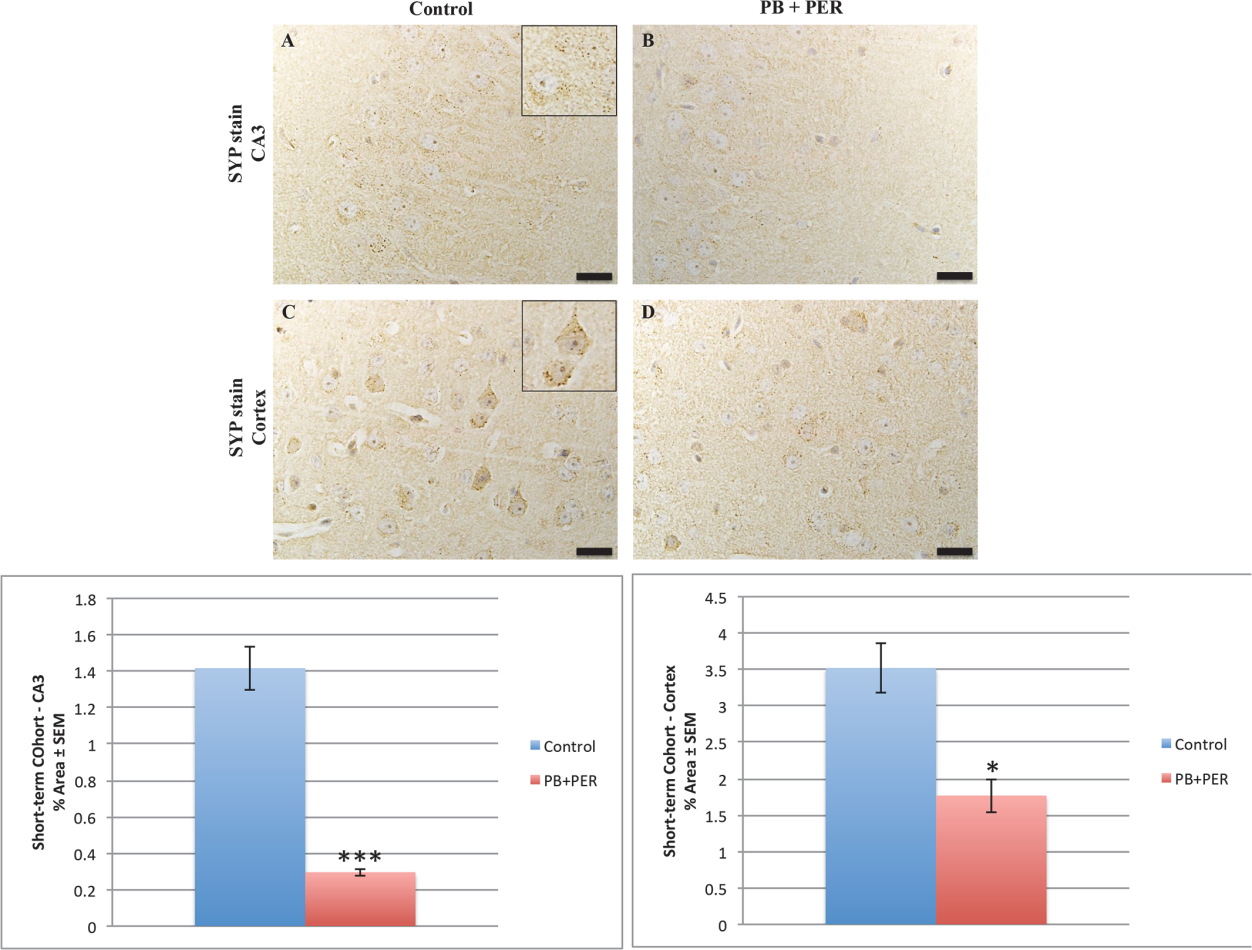
PB + PER reduced SYP staining in the hippocampi and cerebral cortices of exposed mice 18 day post-exposure. Reduced SYP staining was observed in the CA3 region of exposed mice (B) versus controls (A) at 18 days post exposure (Welch’s t-test = 84.08, DF = 1, p < 0.001). In addition, significantly reduced SYP staining was evident in the cerebral cortices of exposed mice (D), as compared to controls (C) (Welch’s t-test = 18.31, DF = 1, p = 0.02). Representative images were taken at 60X magnification (scale bars represent 1 mm). Inset depicts positive SYP staining showing dark brown pre-synaptic vesicles stained within the cell soma (see inset in A and C). Histograms depict the quantification of the SYP stain in the hippocampi and cerebral cortices, as % Area per microscopic field, and error bars show standard error of the mean (SEM).

Neuropathological analyses of the Short-term Cohort revealed significant differences between the exposed and control animals. A reduction in SYP staining was observed when examining the cerebral cortices (Welch’s t-test = 18.31, DF = 1, p = 0.02) and the CA3 regions of exposed and control mice (Welch’s t-test = 84.08, DF = 1, p < 0.001, see Fig. 4).

No differences were detected in GFAP staining in the hippocampi (Welch’s t-test = 0.25, DF = 1, p = 0.81), and the cerebral cortices (Welch’s t-test = 0.57, DF = 1, p = 0.58) of exposed animals (data not shown). Furthermore, no differences were detected in IBA-1 staining when examining the cerebral cortices (Welch’s t-test = 1.18 DF = 1, p = 0.44), and dentate gyri (Welch’s t-test = 1.27, DF = 1, p = 0.34) of exposed animals as compared to controls (S1 Fig.). This was further confirmed by CD45 staining, where no CD45 + cells were detected in either the control or in the exposed animals when examining their cerebral cortices and dentate gyri, whereas CD45 staining was evident in the positive control (sagittal brain sections from the PSAPP mouse model of Alzheimer’s Disease (data not shown)). Nissl and Bielschowsky’s Silver staining did not reveal any gross structural or morphological differences between the two groups (S3 Fig.).

### Neuropathological findings in the Long-term Cohort

**Fig 5:**
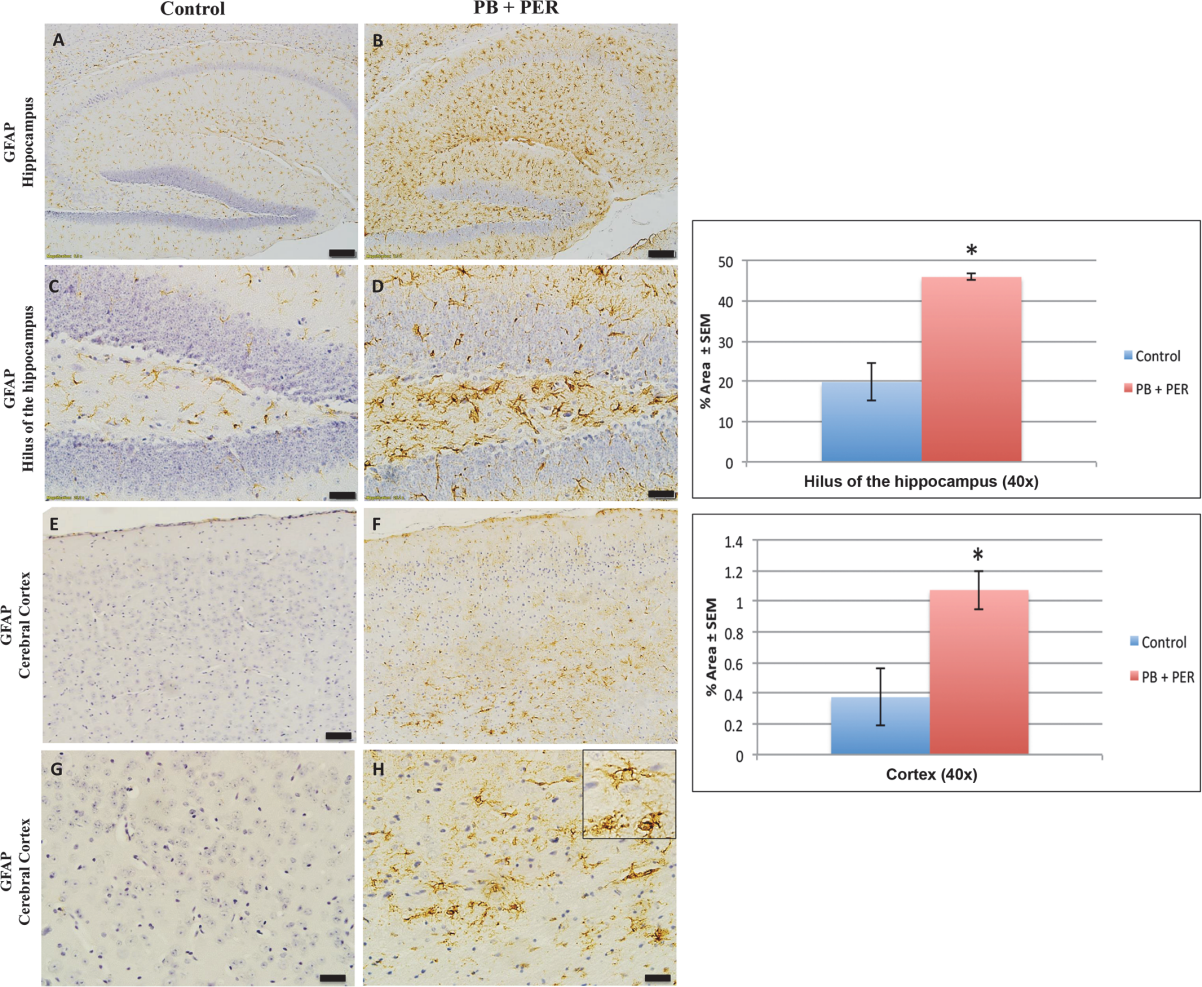
PB + PER exposure altered astrocytic activation in the hili of hippocampi and cerebral cortices of mice. PB + PER exposure significantly increased astrocytic activation in the hili of the hippocampi of exposed mice (B, D) compared to controls (A, C) at 5 months post exposure (Welch t-test = 5.57, DF = 1, p = 0.03). PB + PER exposure significantly increased astrocytic activation in the cerebral cortices (F, H) of Long-term Cohort mice (see inset in H), as compared to controls (E, G) at 5 months post exposure (Welch t-test = 3.14, DF = 1, p = 0.04). Representative images used 10X (A, B, E, F), and 40X (C, D, G, H) objectives, (scale bars represent 100 μm, and 20 μm respectively). Histograms depict the quantification of the GFAP stain in the hili of the hippocampi and cerebral cortices from control and exposed mice, as % Area per microscopic field, and error bars represent standard error of the mean (SEM).

**Fig 6:**
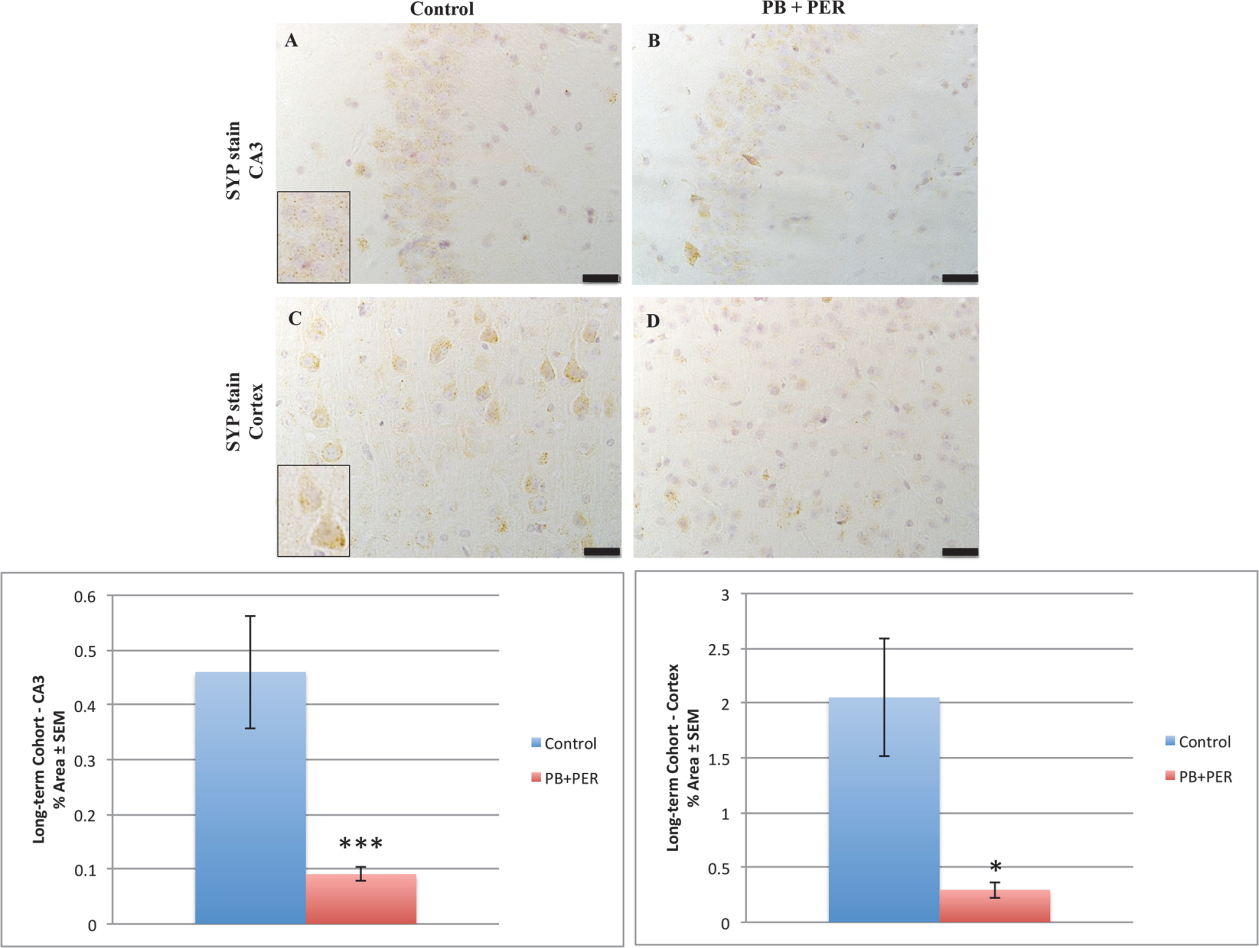
PB + PER exposure decreased SYP staining in the hippocampi and cerebral cortices of exposed mice. Differences in SYP staining was observed in the CA3 region of exposed mice (B) versus controls (A) at 5 months post exposure (Welch’s t-test = 12.7, DF = 1, p < 0.01). In addition, significantly reduced SYP staining was evident in the cerebral cortices of exposed mice (D), as compared to controls (C) (Welch’s t-test = 10.26, DF = 1, p = 0.03). Representative images were taken at 60X magnification (scale bars represent 1 mm). Inset depicts positive SYP staining showing dark brown pre-synaptic vesicles stained within the cell soma (see insets in A and C). Histograms depict the quantification of the SYP stain in the hippocampi, and cerebral cortices, as % Area per microscopic field, and error bars represent standard error of the mean (SEM).

Compared to controls, GW agent exposed mice showed GFAP reactive astrocytes, as characterized by hypertrophied perikarya and processes (“swollen” astrocytes) in the hippocampi, (Welch’s t-test = 5.57, DF = 1, p = 0.03). Furthermore, swollen astrocytes were observed in the cerebral cortices of exposed animals (Welch’s t-test = 3.14, DF = 1, p = 0.04; see Fig. 5). Reduced SYP staining was noticeable when examining the cerebral cortices (Welch’s t-test = 10.26, DF = 1, p = 0.03), and the CA3 areas (Welch’s t-test = 12.7, DF = 1, p < 0.01) of exposed mice (Fig. 6). IBA-1 staining showed no differences between exposed and control mice in the hippocampi (Welch’s t-test = 0.47, DF = 1, p = 0.67), and the cerebral cortices (Welch’s t-test = 0.84, DF = 1, p = 0.44 (S2 Fig.)). In addition, no CD45+ cells were detected in the cerebral cortices and dentate gyri of control or exposed animals (data not shown). Furthermore, exposure to GW agents did not appear to alter neuronal cell morphology, as assessed by Nissl staining of the hippocampi and cerebral cortices of exposed mice compared to controls (S4 Fig.). The majority of cells were devoid of TUNEL staining in all regions examined. There was no indication of positive apoptotic nuclei abnormalities compared to the positive control (DNAse treated brain section), which has a dark brown staining (S4 Fig.). Similarly, the majority of cells in the hippocampi of exposed mice appeared to retain normal cell morphology and were free from damaged and swollen axons and degenerated neurons (S4 Fig.) when compared to a positive control (sagittal brain sections from the PSAPP mouse model of Alzheimer’s disease).

### Brain ACh levels

**Fig 7:**
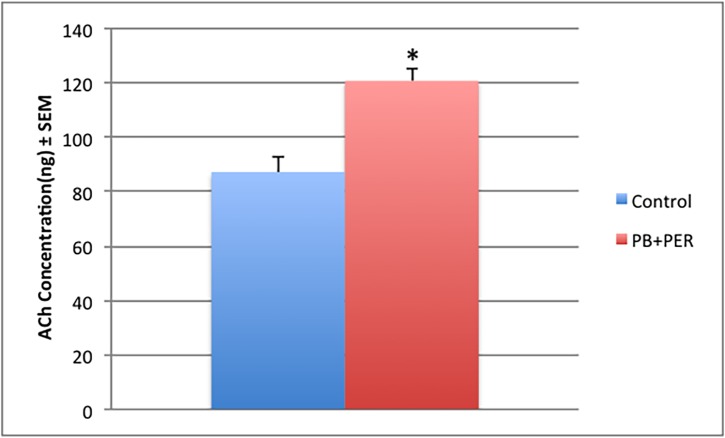
ACh levels were increased in the CNS of exposed mice. Mass Spectrometric (MS) analysis of ACh levels in brain homogenates from exposed and control animals at 5 months post acute exposure showed a significant effect of GW agent exposure on ACh levels (F = 20.5, *P* < 0.0001). There was a 1.40-fold increase in ACh in PB and PER exposed mice as compared to controls. Error bars represent standard error of the mean (SEM).

Mass Spectrometric analysis of ACh levels in the PBS fraction of total brain homogenate showed a significant increase of 1.40-fold in exposed mice as compared to controls in the Long-term Cohort (F = 20.5, *p* < 0.0001; see Fig. 7). No such differences were observed in the Short-term Cohort (F = 0.48, p = 0.87; data not shown).

## Discussion

It has been over two decades since the 1990–1991 Persian Gulf War conflict, and GWI remains an untreatable illness, in part due to the multifaceted etiology and presentation of the illness, which derives from the complexity of the underlying pathobiology. In this study, we conducted a neurobehavioral examination over a period of 5 months post exposure, as well as an extensive neuropathological investigation of the responses associated with GW agent exposure at short-term and long-term time points in the current GW mouse model. We successfully translated our previous model of GW agent (PB+PER) exposure from the CD1 strain [14] to the more commonly used C57BL6/J strain, as this strain is widely used in preclinical research. In addition, this strain has many readily available transgenic and knockdown models, and thus will facilitate future evaluation of the effects of different genetic influences, which may well have relevance for influencing GWI clinical presentation.

In the current study, we used an acute (10 days) exposure to PB and PER, as we previously mentioned we have successfully translated our GW mouse model from a CD1 stain to a more widely used C57BL6/J strain, in order to further characterize this model. Combined exposure to these agents have been implicated in the memory deficits associated with GWI [1]. Several studies consistently show that in rodents, combined exposure to PB and PER alone, or in combination with DEET and stress, results in neurobehavioral deficits (i.e. anxiety, mood and cognitive impairment) that are similar to symptoms reported by veterans with GWI [1,19–21,24,32–34]. In addition, other GW agents have also been proposed, such as depleted uranium, multiple vaccinations against anthrax and botulinum, as well as exposure to low levels of nerve gas agents [10,12,29,31,45,48,52]. However, clinical support is much stronger for a possible causative role of combined exposure to PB and pesticides (i.e. PER) in the etiology of GWI [21,24,32,35–40]. We have therefore focused on characterizing the neurobehavioral and neuropathological consequences of combined exposure to PB and PER, until additional clinical data become available for the other exposures. Our studies show that acute combined exposure to PB and PER results in long-term cognitive deficits, which are associated with astroglia activation, pre-synaptic vesicle loss and a perturbation of the CNS cholinergic system.

Using the Barnes Maze test, we examined learning, short- and long-term memory at a range of immediate and chronic post-exposure time-points. We observed an impairment of long-term memory formation in exposed mice without any deficits in learning or short-term memory.

A number of studies show that long-term memory formation is a hippocampus dependent event facilitated by hippocampal working memory recruitment [41–44]. It has been demonstrated that the hippocampus not only plays a role in long-term memory encoding, but is also important for working memory as well, in particular when multiple items are being processed [45]. Several independent studies show possible hippocampal dysfunction in veterans with GWI [46,47]. Studies have shown brain alterations in ill GW veterans, exemplified by changes in the regional cerebral blood flow (rCBF) in the hippocampi of patients with Gulf War syndromes 1 (impaired cognition), 2 (confusion-ataxia) and 3 (central neuropathic pain), as compared to controls. Challenge with physostigmine significantly decreased hippocampal regional cerebral blood flow (rCBF) in control participants and veterans with syndrome 1, but significantly increased rCBF in the right hippocampus of veterans with syndrome 2, and in both hippocampi of the veterans with syndrome 3, suggesting chronic alteration of hippocampal blood flow [46]. In particular, a brain imaging study showed that compared to healthy controls, veterans with GWI had significantly smaller hippocampal volume, which corresponded with lower scores on neuropsychological tests for immediate and delayed verbal and visual retrieval memory [8,48]. As such, deficits in long-term memory formation may be an indication of damage to the hippocampus and require further examination. More specifically, the aspect of hippocampal neurogenesis and the interplay between short- and long-term memory formations require further investigation in subsequent studies.

Immunohistochemical analyses identified astroglial activation in the hippocampi and cerebral cortices of exposed mice at 5 months post-exposure. We, and others, have previously reported an increase in astrogliosis in mouse models of GW agent exposure [21,24,34,49]. The increased astrogliosis identified in the Long-term Cohort is of particular interest, as astrocyte dysfunction has been linked to altered brain function in veterans with GWI [50]. For instance, H^1^ MRS spectroscopy conducted by Rayhan and colleagues show that a proportion of GWI subjects had elevated prefrontal lactate that was predictive of exercise induced cognitive dysfunction. Astrocytes produce lactate from anaerobic glycolysis that is then used by neurons as metabolic fuel [39]. Suzuki et al. showed that in the rodent hippocampus, astrocytic glycogen breakdown and lactate release are essential for long-term memory formation and for the maintenance of long-term potentiation (LTP) of synaptic strength [51]. Consequently, based on the recent reports by Rayihan and colleagues, we therefore hypothesize that a dysfunctional neuronal-astrocyte relationship in GWI might underlie some of the symptoms present in the patient population [50,52].

While examining the cerebral cortices and dentate gyri of exposed mice, we were not able to detect activated microglia across all time-points examined in this study. Our findings using IBA-1 staining revealed the presence of small and ramified resting microglial cells. We further confirmed the lack of activated microglia using a CD45 marker. Our observations are in accordance with our previous studies and those of others that showed either a lack of or marginal microglial activation [20,34,49], but given the potentially transient nature of microgliosis [53–55], it is possible that the time points we examined in our current study flanked a period of microgliosis which had subsided by the 5 month time point. We acknowledge that there are limitations regarding our current neuropathological work, as immersion-fixed brain samples were used in this study: the histological data will need further confirmation in future studies using perfusion-fixed tissues.

We observed a reduction in SYP (a marker of pre-synaptic vesicles) staining in the hippocampi of exposed mice at the long-term time point and a reduction in the cerebral cortices of exposed mice at both short- and long-term time points. Synaptic abnormalities in the hippocampus correlate with the severity of neuropathology and memory deficit in individuals suffering with neurological diseases [56–60]. Measurements of synaptic vesicle proteins and GFAP have been previously used to characterize the temporal and regional patterns of neuronal and glial responses to injury [61–63]. Altered synaptic morphology, progressive loss of synapses, and glial cell activation are considered characteristic hallmarks of cognitive decline [64,65]. Furthermore, much interest centers on the role of astrocytes in the modulation of synaptic transmission and their involvement in the induction of plasticity, such as long-term potentiation and long-term depression [66]. Collectively, the presence of astroglial activation, together with pre-synaptic loss in GW agent exposed mice, may be indicative of reduced neuroglial support of synapses. However, further work is needed to decipher the mechanisms underpinning the observed neuropathological changes.

To the best of our knowledge, the current study is the first report to demonstrate increased levels of ACh in the brains of mice at a late time point post-exposure to GW agents, which may indicate a disturbed homeostatic imbalance of the cholinergic system. Furthermore, organophosphorous insecticides, some of which have been attributed to the etiology behind GWI, are potent inhibitors of acetylcholinesterase (AChE) activity [1,24,67,68]. Exposure to these compounds has been shown to induce both acute toxicity and long-term neurological deficits [69–73]. The research focus regarding these compounds has been on their ability to inhibit AChE, and how this leads to hyperstimulation of cholinergic systems. The ability of organophosphates to inhibit AChE has led to the hypothesis that they exert their neurotoxic effects by increasing ACh concentrations, leading to overstimulation of cholinergic receptors and thereby inducing seizure activity and excitotoxic neuronal death [74]. Since neuronal cells receive ACh input from the basal forebrain region and express muscarinic ACh receptors (mAChR) and nicotinic ACh receptors (nAChR) [75,76], it is possible that initially, or at a short time point, the inhibition of AChEs may lead to an increase in both the level of and action duration of ACh [77,78]. In addition, it has been shown that co-exposure to PB, DEET, and PER resulted in increased ligand binding for m2 muscarinic acetylcholine receptor in the cortex and significantly increased ligand binding for nicotinic acetylcholine receptor [19]. Thus, we hypothesize that excessive synaptic ACh levels can lead to the down-regulation or desensitization of these receptors, where ACh may take on an inhibitory role, causing further nervous system depression at a late time point post-exposure. However, further work is needed to decipher the exact mechanisms underpinning our observations. Overall, these results suggest that exposure to GW agents may be attributed to the impaired cholinergic function observed in GWI and may in part contribute to deficits observed in long-term memory formation.

## Conclusions

Overall, our findings from the characterization of a mouse model of GW agent exposure at short-and long-term time points post-exposure have provided insight into the multi-symptom presentation of GWI. We believe that our model will be instrumental in enabling further research into the biological pathways that are modulated at late time points after GW agent exposure, which is the time point of most relevance to the GWI patient population who received their pathogenic exposures more than 23 years ago. In addition, in subsequent studies we plan to characterize later post exposure time points in this mouse model, as we have now successfully translated our findings from the CD1 stain to the C57BL6/J stain. We anticipate that our subsequent studies will provide us with a more lifetime picture regarding the long-lasting consequences of GW agent exposure. This may then lead to the identification of potential therapeutic targets for our GWI patient population, which are critically in demand.

## Supporting Information

S1 Fig PB + PER exposure did not alter microglial levels in hippocampi and cortices of mice 18 days post-exposure. The IBA-1 stain showed no differences between exposed (B, D) and control (A, C) mice in the hippocampi (Welch’s t-test = 1.27, DF = 1, p = 0.3) and the cerebral cortices of exposed (F, H) and control (E, G) animals (Welch’s t-test = 1.18 DF = 1, p = 0.44). Representative images used 10X (A, B, E, F), and 40X (C, D, G, H) objectives, scale bars represent 100 μm, and 20 μm, respectively. Histograms depict the quantification of the IBA-1 stain in the hippocampi and cerebral cortices from control and exposed mice as % Area per microscopic field, and error bars show standard error of the mean (SEM).
(TIF)

S2 Fig PB + PER exposure did not alter microglial levels in hippocampi and cortices of mice 5 months post-exposure. The IBA-1 stain showed no differences between exposed (B, D) and control (A, C) mice in the hippocampi (Welch’s t-test = 0.47, DF = 1, p = 0.67) and the cerebral cortices of exposed (F, H) and control (E, G) animals (Welch’s t-test = 0.84, DF = 1, p = 0.44). Representative images used 10X (A, B, E, F), and 40X (C, D, G, H) objectives, scale bars represent 100 μm, and 20 μm, respectively. Histograms depict the quantification of the IBA-1 stain in the hippocampi and cerebral cortices from control and exposed mice as % Area per microscopic field, and error bars show standard error of the mean (SEM).
(TIF)

S3 Fig No alterations in cell morphology detected 18 days post exposure to PB+PER. Nissl staining revealed no gross morphological changes in nuclei/cell body of pyramidal neurons post exposure to PB+PER (A-D). Similarly, the majority of cells in the hippocampi and cerebral cortices of PB+PER exposed mice as compared to controls (E-H) were free from damaged and swollen axons and degenerated neurons when compared to a positive control (PSAPP mouse model of Alzheimer’s disease; see inset in F). Representative images were taken at 40X magnification (scale bar represents 20 μm).
(TIF)

S4 Fig No alterations in cell morphology detected 5 months post exposure to PB+PER. Nissl staining revealed no gross morphological changes in nuclei/cell body of pyramidal neurons post exposure to PB+PER (A-D). TUNEL was used to detect apoptotic cells. The majority of cells were devoid of TUNEL staining in all regions examined (E-H), there was no indication of positive apoptotic nuclei abnormalities compared to positive controls (DNAse treated brain section), which have a dark brown staining (see inset in F). Similarly, the majority of cells in the hippocampi and cerebral cortices of PB+PER exposed mice as compared to controls (I-L) were free from damaged and swollen axons and degenerated neurons when compared to a positive control (PSAPP mouse model of Alzheimer’s disease; see inset in J). Representative images were taken at 40X magnification (scale bar represents 20 μm).
(TIF)
