# Supplementary figures and images for: Gulf War Agent Exposure Causes Impairment of Long-Term Memory Formation and Neuropathological Changes in a Mouse Model of Gulf War Illness
Source: PLoS One. 2015 Mar 18;10(3):e0119579. doi: 10.1371/journal.pone.0119579 (PMC4364893; doi:10.1371/journal.pone.0119579)

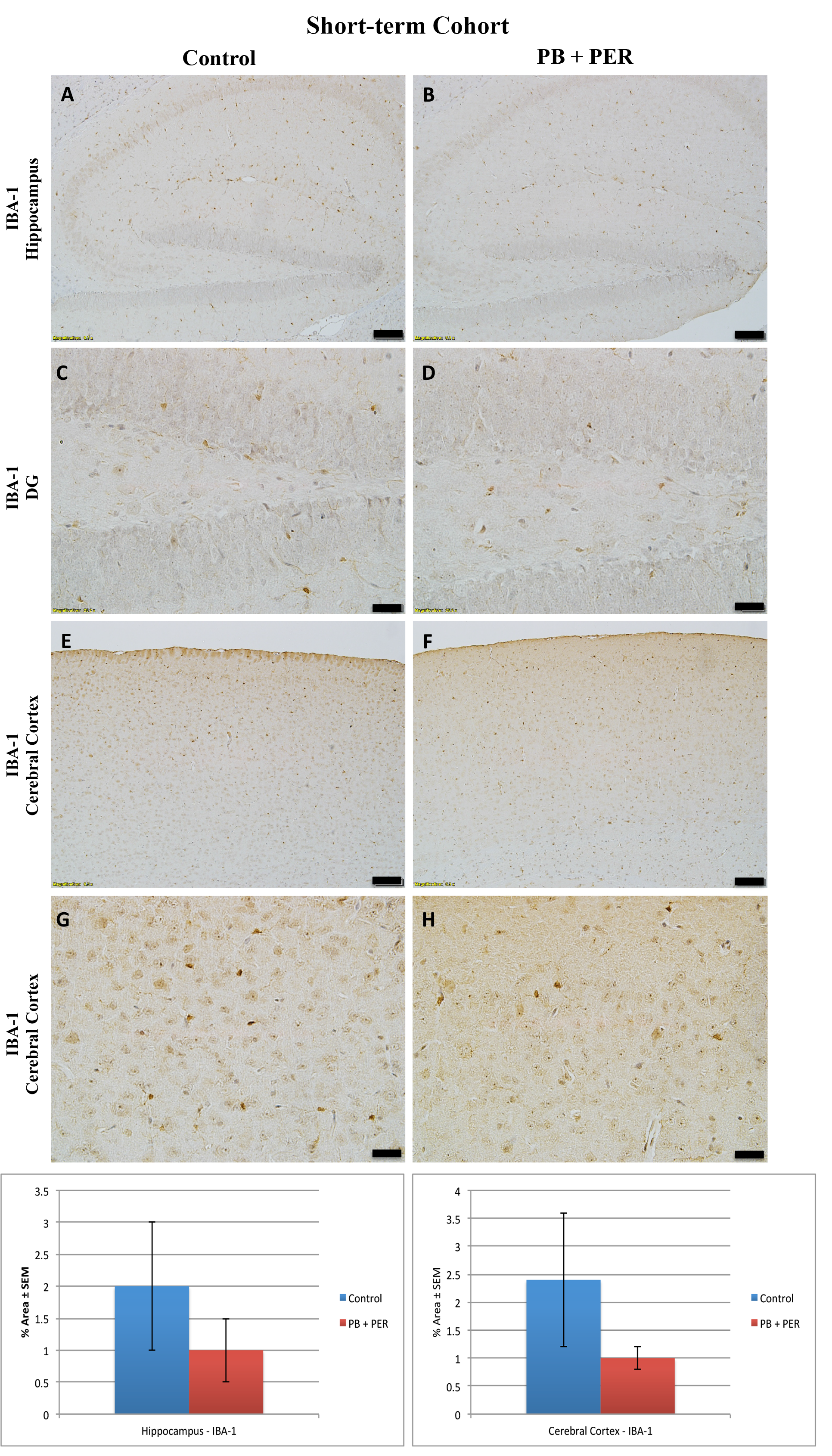

Supplement: S1 Fig — The IBA-1 stain showed no differences between exposed (B, D) and control (A, C) mice in the hippocampi (Welch’s t-test = 1.27, DF = 1, p = 0.3) and the cerebral cortices of exposed (F, H) and control (E, G) animals (Welch’s t-test = 1.18 DF = 1, p = 0.44). Representative images used 10X (A, B, E, F), and 40X (C, D, G, H) objectives, scale bars represent 100 μm, and 20 μm, respectively. Histograms depict the quantification of the IBA-1 stain in the hippocampi and cerebral cortices from control and exposed mice as % Area per microscopic field, and error bars show standard error of the mean (SEM). (TIF) [file pone.0119579.s001.tif]

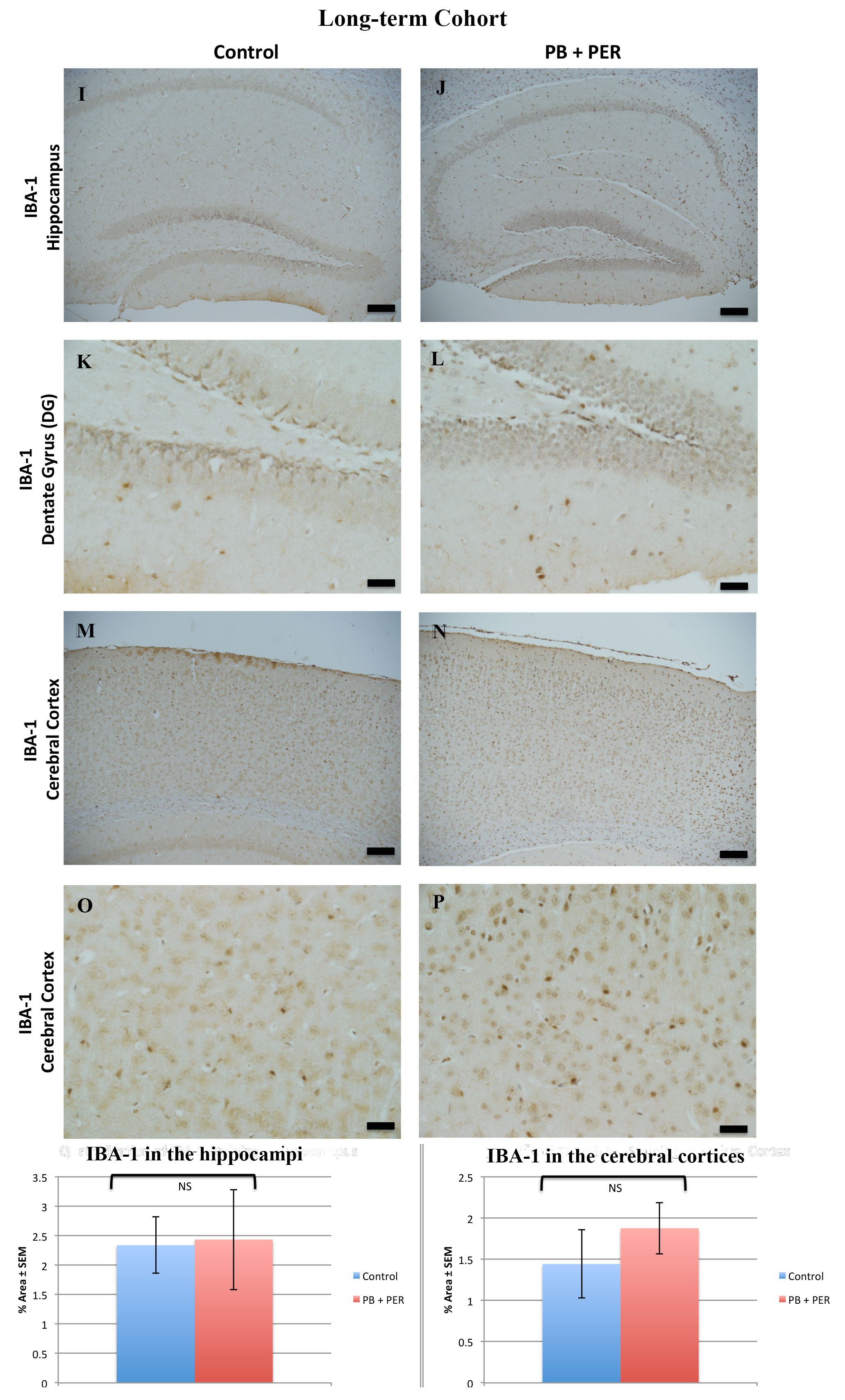

Supplement: S2 Fig — The IBA-1 stain showed no differences between exposed (B, D) and control (A, C) mice in the hippocampi (Welch’s t-test = 0.47, DF = 1, p = 0.67) and the cerebral cortices of exposed (F, H) and control (E, G) animals (Welch’s t-test = 0.84, DF = 1, p = 0.44). Representative images used 10X (A, B, E, F), and 40X (C, D, G, H) objectives, scale bars represent 100 μm, and 20 μm, respectively. Histograms depict the quantification of the IBA-1 stain in the hippocampi and cerebral cortices from control and exposed mice as % Area per microscopic field, and error bars show standard error of the mean (SEM). (TIF) [file pone.0119579.s002.tif]

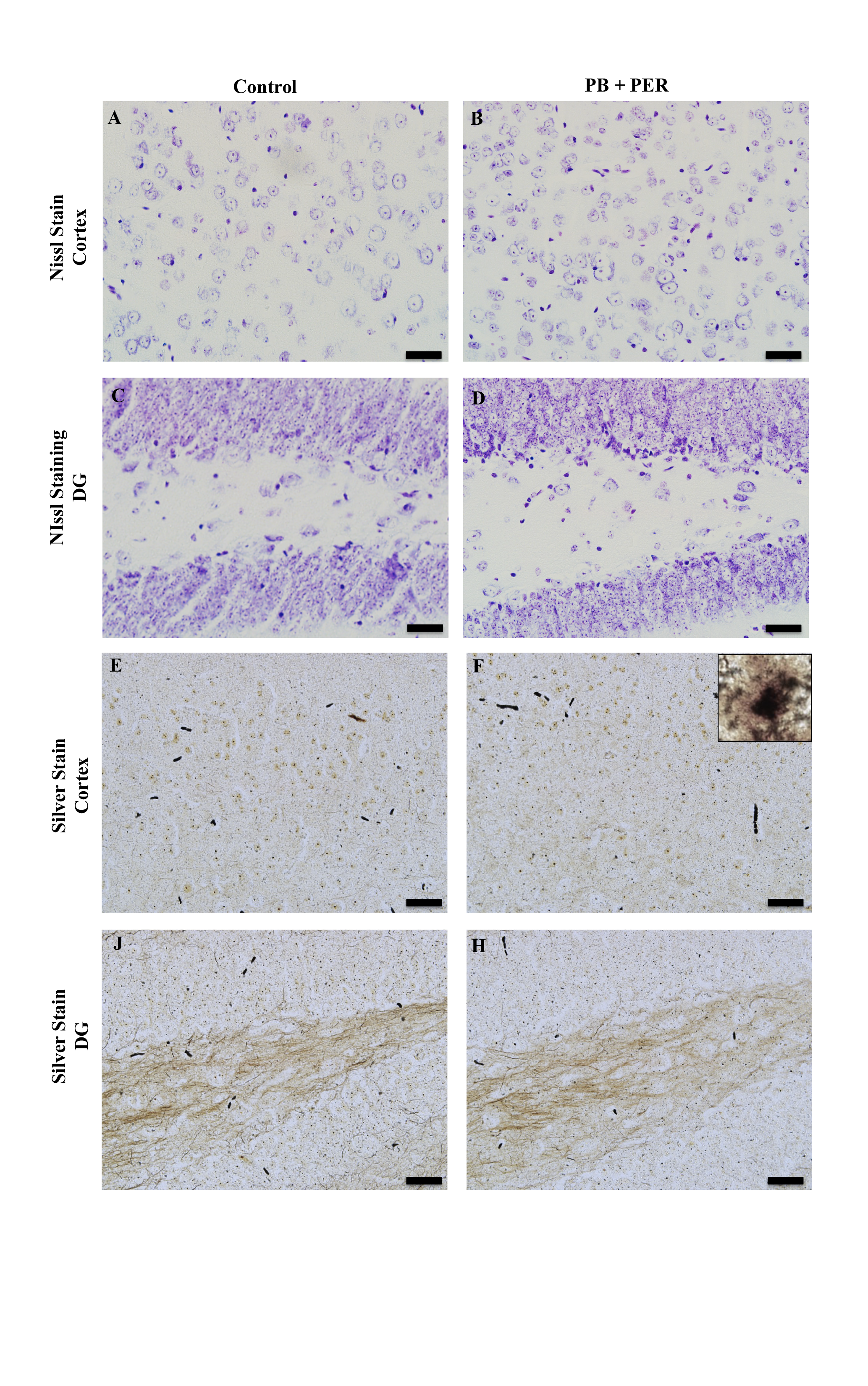

Supplement: S3 Fig — Nissl staining revealed no gross morphological changes in nuclei/cell body of pyramidal neurons post exposure to PB+PER (A-D). Similarly, the majority of cells in the hippocampi and cerebral cortices of PB+PER exposed mice as compared to controls (E-H) were free from damaged and swollen axons and degenerated neurons when compared to a positive control (PSAPP mouse model of Alzheimer’s disease; see inset in F). Representative images were taken at 40X magnification (scale bar represents 20 μm). (TIF) [file pone.0119579.s003.tif]

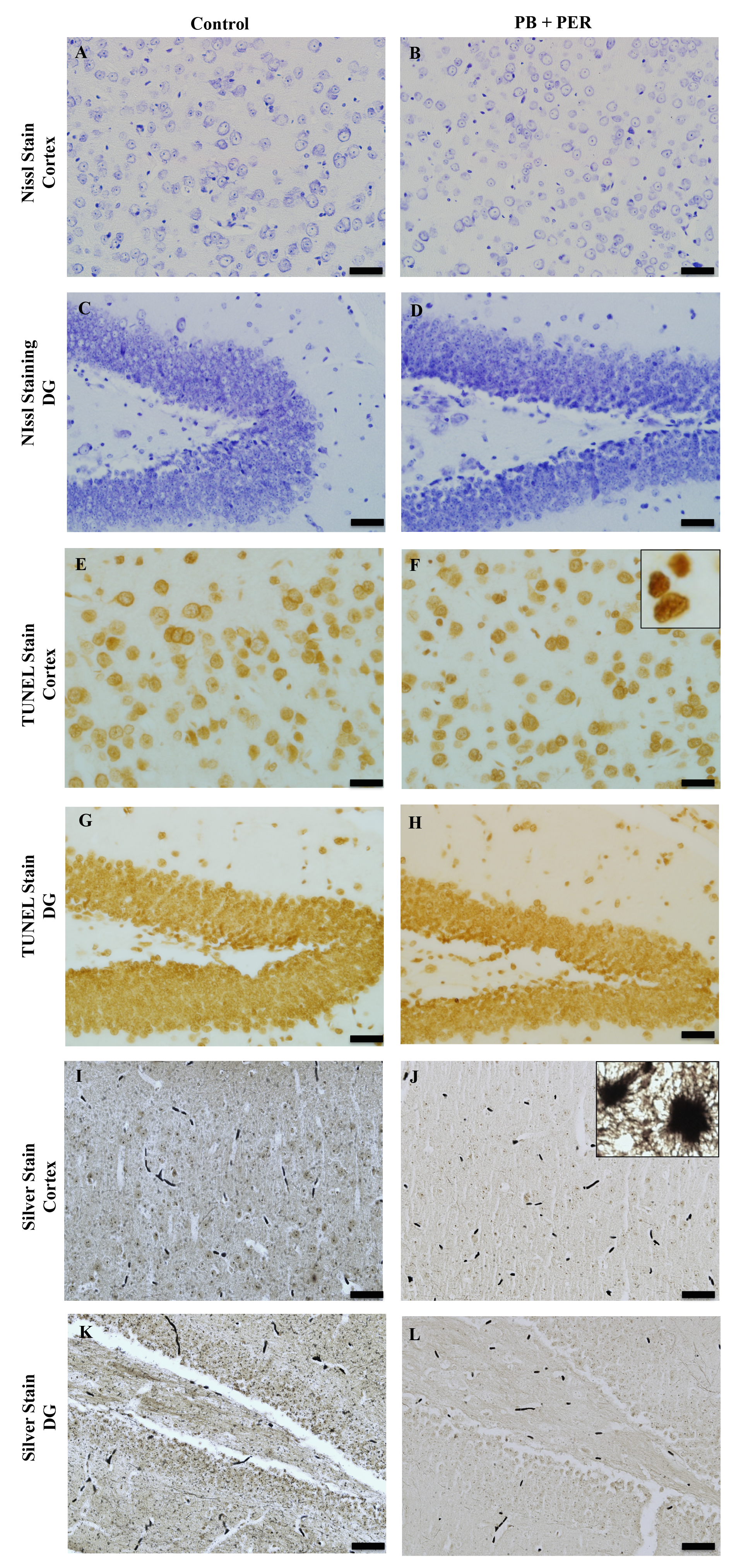

Supplement: S4 Fig — Nissl staining revealed no gross morphological changes in nuclei/cell body of pyramidal neurons post exposure to PB+PER (A-D). TUNEL was used to detect apoptotic cells. The majority of cells were devoid of TUNEL staining in all regions examined (E-H), there was no indication of positive apoptotic nuclei abnormalities compared to positive controls (DNAse treated brain section), which have a dark brown staining (see inset in F). Similarly, the majority of cells in the hippocampi and cerebral cortices of PB+PER exposed mice as compared to controls (I-L) were free from damaged and swollen axons and degenerated neurons when compared to a positive control (PSAPP mouse model of Alzheimer’s disease; see inset in J). Representative images were taken at 40X magnification (scale bar represents 20 μm). (TIF) [file pone.0119579.s004.tif]
